# Combining FoxP3 and Helios with GARP/LAP markers can identify expanded Treg subsets in cancer patients

**DOI:** 10.18632/oncotarget.7334

**Published:** 2016-02-11

**Authors:** May Abd Al Samid, Belal Chaudhary, Yazan S. Khaled, Basil J. Ammori, Eyad Elkord

**Affiliations:** ^1^ Biomedical Research Centre, School of Environment and Life Sciences, University of Salford, Manchester, United Kingdom; ^2^ College of Medicine and Health Sciences, United Arab Emirates University, Al Ain, United Arab Emirates; ^3^ Institute of Cancer Sciences, University of Manchester, Manchester, United Kingdom

**Keywords:** regulatory T cells, markers, GARP/LAP, FoxP3, Helios

## Abstract

Regulatory T cells (Tregs) comprise numerous heterogeneous subsets with distinct phenotypic and functional features. Identifying Treg markers is critical to investigate the role and clinical impact of various Treg subsets in pathological settings, and also for developing more effective immunotherapies. We have recently shown that non-activated FoxP3^−^Helios^+^ and activated FoxP3^+/–^Helios^+^ CD4^+^ T cells express GARP/LAP immunosuppressive markers in healthy donors. In this study we report similar observations in the peripheral blood of patients with pancreatic cancer (PC) and liver metastases from colorectal cancer (LICRC). Comparing levels of different Treg subpopulations in cancer patients and controls, we report that in PC patients, and unlike LICRC patients, there was no increase in Treg levels as defined by FoxP3 and Helios. However, defining Tregs based on GARP/LAP expression showed that FoxP3^−^LAP^+^ Tregs in non-activated and activated settings, and FoxP3^+^Helios^+^GARP^+^LAP^+^ activated Tregs were significantly increased in both groups of patients, compared with controls. This work implies that a combination of Treg-specific markers could be used to more accurately determine expanded Treg subsets and to understand their contribution in cancer settings. Additionally, GARP^−/+^LAP^+^ CD4^+^ T cells made IL-10, and not IFN-γ, and levels of IL-10-secreting CD4^+^ T cells were elevated in LICRC patients, especially with higher tumor staging. Taken together, our results indicate that investigations of Treg levels in different cancers should consider diverse Treg-related markers such as GARP, LAP, Helios, and others and not only FoxP3 as a sole Treg-specific marker.

## INTRODUCTION

Regulatory T cells (Tregs) are immunosuppressive cells with key roles in immune tolerance and immune dysregulation in pathological settings including inflammation, autoimmunity and cancer [1]. In cancers, Tregs accumulate in peripheral tissues and tumors where, in conjunction with other immunosuppressive cells, they inhibit tumor specific immune responses and contribute to the development of a tolerogenic tumor microenvironment enabling immune evasion [2, 3]. Elevated Treg levels have been reported to correlate with tumor progression, impaired T cell functionality and negative prognosis in different cancers [4–7]. Given their role in immune evasion and poor clinical outcomes, Tregs have become an important target for novel cancer immunotherapies [3, 8–10].

In recent years, it has become increasingly clear that Tregs comprise diverse subsets with distinct phenotypic and functional features [11–15]. Understanding the role and contribution of specific Treg subsets is critical to harnessing the potential of different therapeutic modalities. Tregs are generally divided into thymic-derived Tregs (tTregs) and peripheral-induced Tregs (pTregs), traditionally defined by expression of the forkhead box P3 transcription factor (FoxP3) and IL-2 receptor alpha chain (CD25). In addition, two FoxP3^−^ pTreg subsets have been identified; Tr1 and Th3 cells. Significant efforts have been made into identifying effective markers for Treg subset identification, isolation and therapeutic manipulation [4, 15, 16]. Both CD25 and FoxP3 can be up-regulated on non-suppressive Teff and activated T cells, while FoxP3 as an intracellular marker does not allow Treg isolation [17]. Promising Treg markers include the late-stage Treg activation markers, glycoprotein A repetitions predominant (GARP) and latency-associated peptide (LAP), and the Ikaros zinc finger transcription factor Helios.

Helios has been suggested to play important roles in immune regulation by repressing pro-apoptotic genes in Tregs, contributing to the development of follicular Tregs, and enhancing Treg function in cooperation with FoxP3 [18, 19]. Despite its seemingly ubiquitous expression, it is accepted that Helios can define highly suppressive Treg subsets in various settings. FoxP3^+/–^Helios^+^ Tregs are significantly expanded in the peripheral blood and at tumor sites of various cancers, and have been reported to exhibit enhanced *in vitro* suppressive activity [20, 21].

GARP and LAP are well-characterized late-stage Treg activation markers, and they contribute directly to a contact-dependent TGF-β-mediated suppressive mechanism in Tregs [22, 23]. LAP is a propeptide that binds non-covalently with transforming growth factor beta (TGF-β) forming an inactive latent LAP-TGF-β complex, and TGF-β is cleaved from the latent complex releasing active TGF-β [22]. LAP has been utilized to isolate highly suppressive Tregs in *in vitro* expansion cultures and also from the peripheral blood of cancer patients following CTLA-4 immunotherapy [24, 25]. GARP is a transmembrane protein that plays a critical role in the formation and expression of LAP-TGF-β complexes by anchoring the complexes to the cell membrane [23].

We have recently shown that non-activated FoxP3^−^Helios^+^ and activated FoxP3^+/–^Helios^+^ CD4^+^ T cells isolated from the peripheral blood of healthy donors co-express GARP and LAP [26]. In the current study we report similar observations in T cells isolated from the peripheral blood of patients with pancreatic cancer (PC) and patients with liver metastases from colorectal cancer (LICRC). In addition, we show that FoxP3^+/–^Helios^+^GARP^+^LAP^+^ activated Treg subsets are expanded in PC and LICRC patients, compared with healthy donors. We also report that CD4^+^GARP^+/–^LAP^+^ T cells make IL-10 but not IFN-γ, and they are increased in LICRC patients.

## RESULTS

### LAP is expressed significantly higher than GARP on activated CD4^+^ T cells in healthy donors and pancreatic cancer patients

**Figure 1:**
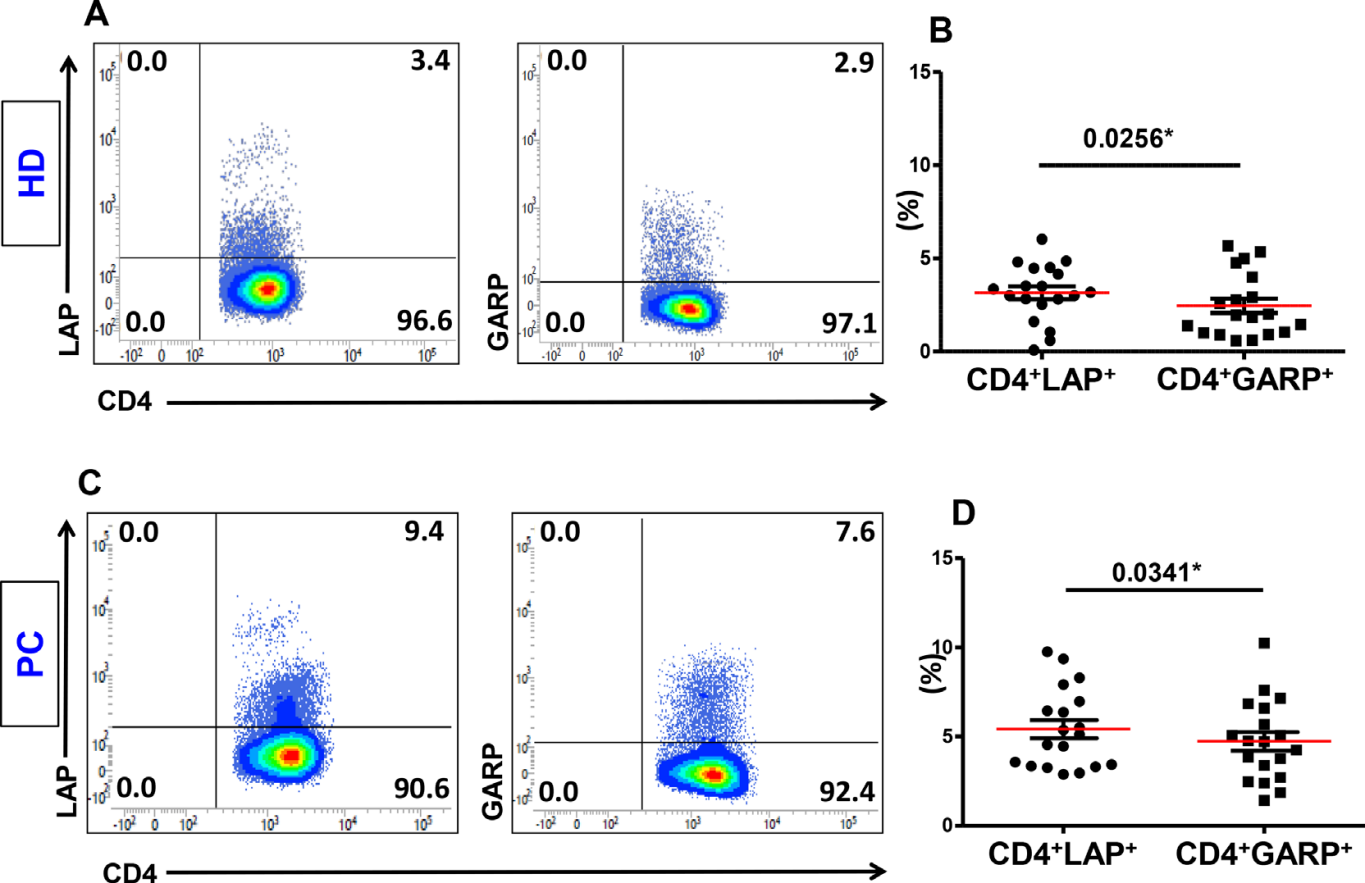
Expression of LAP or GARP on activated CD4^+^ T cells PBMCs from 19 healthy donors (HD) and 19 pancreatic cancer (PC) patients were activated by plate-bound anti-CD3/28 followed by staining for LAP and GARP. Representative flow cytometric plots showing LAP (first plots) or GARP (second plots) expression on CD3^+^CD4^+^ T cells isolated from HD (**A**) and PC patients (**C**). Scatter plots show the mean percentages ± SEM of CD4^+^LAP^+^ T cells compared with CD4^+^GARP^+^ T cells in activated PBMCs isolated from HD (**B**) and PC patients (**D**).

Peripheral blood samples were collected from PC and LICRC patients and chronic pancreatitis (CP) and Healthy donor (HD) controls. as detailed in Table 1. We first compared the expression of LAP and GARP, as markers of activated Tregs, on CD4^+^ T cells isolated from the peripheral blood of HD and PC patients. LAP and GARP were expressed at low levels on CD4^+^ T cells in the steady state (< 1% for HD and < 2% for PC patients, data not shown). Following *in vitro* activation with anti-CD3/28, both GARP and LAP were significantly up regulated on CD4^+^ T cells, as expected. However, expression of LAP was higher than GARP on CD4^+^ T cells. This difference was significant in healthy donors (LAP: 3.15 ± 0.35% vs. GARP 2.46 ± 0.39%; Figure 1A and 1B) and PC patients (LAP: 5.41 ± 0.51% vs. GARP: 4.73 ± 0.52%; Figure 1C and 1D).

**Table 1:** Characteristic features of study subpopulations

	PC	CP		CRC
**Number**	*n =* 20	*n =* 9		*n =* 11
**Age (median)**	62 (47–87)*	54 (31–84)*		73 (71–83)*
**Gender (Male: Female)**	13:7	5:4		8:3
**TNM stage**				
**I**	0	-		1
**II**	4	-		5
**III**	1	-		5
**IV**	15	-		-
**Tumor size (cm)**	2.9 (1.9–5.5)*			4.2 (1–13)*
**Preoperative CA19–9 (0–37 U/ml)**	371 (77–1230)*	49		63.9 (1–169)*
**Preoperative CEA (< 2.5 ng/ml)**	5 (5–13)*	-		29.5 (1–144)*
**Tumor site**				
Head of pancreas	18	-	Right-sided origin	7
Body of pancreas	0	-	Left-sided origin	3
Tail of pancreas	2	-	Others	1
**Histological grade**				
Well/moderate	9	-		11
Poor/undifferentiated	11	-		0

Abbreviations: PC: pancreatic cancer; CP: chronic pancreatitis; CRC: colorectal; CA19–9: cancer antigen 19–9; CEA: carcinoembryonic antigen. *Data shown represent median (range).

### Levels of FoxP3^+^LAP^−^, FoxP3^+^LAP^+^ and FoxP3^−^LAP^+^ Treg subsets in cancer patients and controls

**Figure 2:**
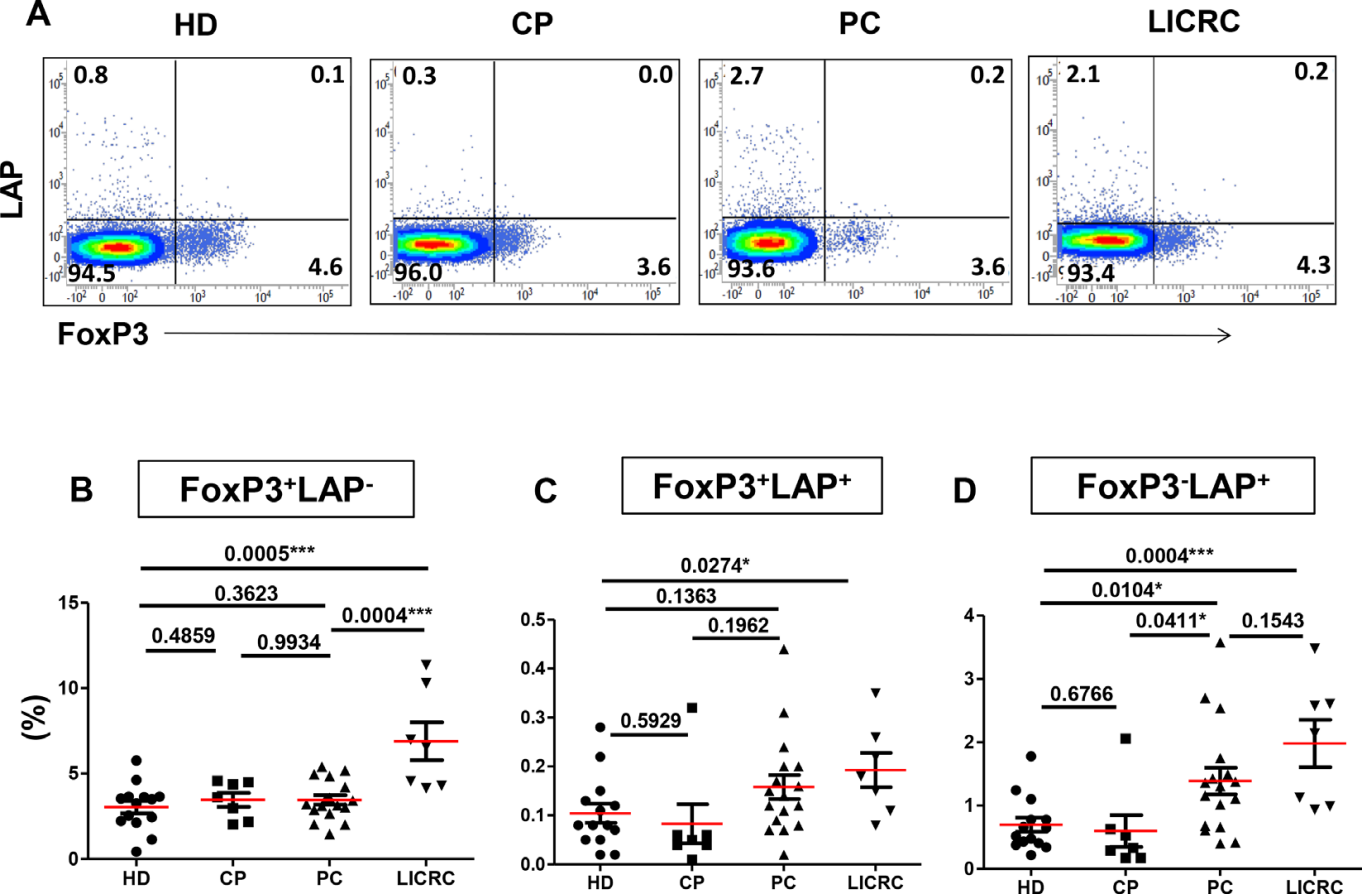
Comparisons between healthy donors and patients for the expression of LAP on non-activated FoxP3^+/–^ T cell subsets Thawed PBMCs isolated from 14 healthy donors (HD), 7 chronic pancreatitis (CP), 17 pancreatic cancer (PC), and 7 liver metastases from colorectal cancer (LICRC) patients were stained for surface and intracellular markers. (**A**) Representative flow cytometric plots showing FoxP3 expression against LAP, gated on CD3^+^CD4^+^T cells from healthy donors and patients. Scatter plots show the mean percentages ± SEM of FoxP3^+^LAP^−^ (**B**), FoxP3^+^LAP^+^ (**C**) and FoxP3^−^LAP^+^ T cells (**D**).

We then analyzed FoxP3 and LAP co-expression on non-activated CD4^+^ T cells (Figure 2). We found that LAP was co-expressed with FoxP3 at very low levels (< 0.2%) on non-activated CD4^+^ Tregs from HD, CP, PC and LICRC. This is consistent with our recent finding in healthy donors [26].

Most studies determine Treg levels based on FoxP3 expression. Defining Tregs based on FoxP3 expression alone, there was no significant increase in the FoxP3^+^LAP^−^ Treg subset in PBMCs from PC (3.46 ± 0.28%), compared to HD (3.04 ± 0.36%) and CP (3.46 ± 0.41%) (Figure 2B). However, the FoxP3^+^LAP^−^ Treg subset was significantly increased in PBMCs from LICRC patients (6.90 ± 1.11%), compared with PC patients and HD (Figure 2B). A very small population (< 0.2%) of double-positive FoxP3^+^LAP^+^ T cells was detected in all samples, but it was higher in LICRC patients compared with HD (Figure 2C). Interestingly, defining Tregs as FoxP3^−^LAP^+^, there were significant increases both in LICRC (1.98 ± 0.37%) and PC (1.39 ± 0.21%) samples, compared with HD (0.70 ± 0.11%) and CP controls (0.60 ± 0.25%) (Figure 2D).

**Figure 3:**
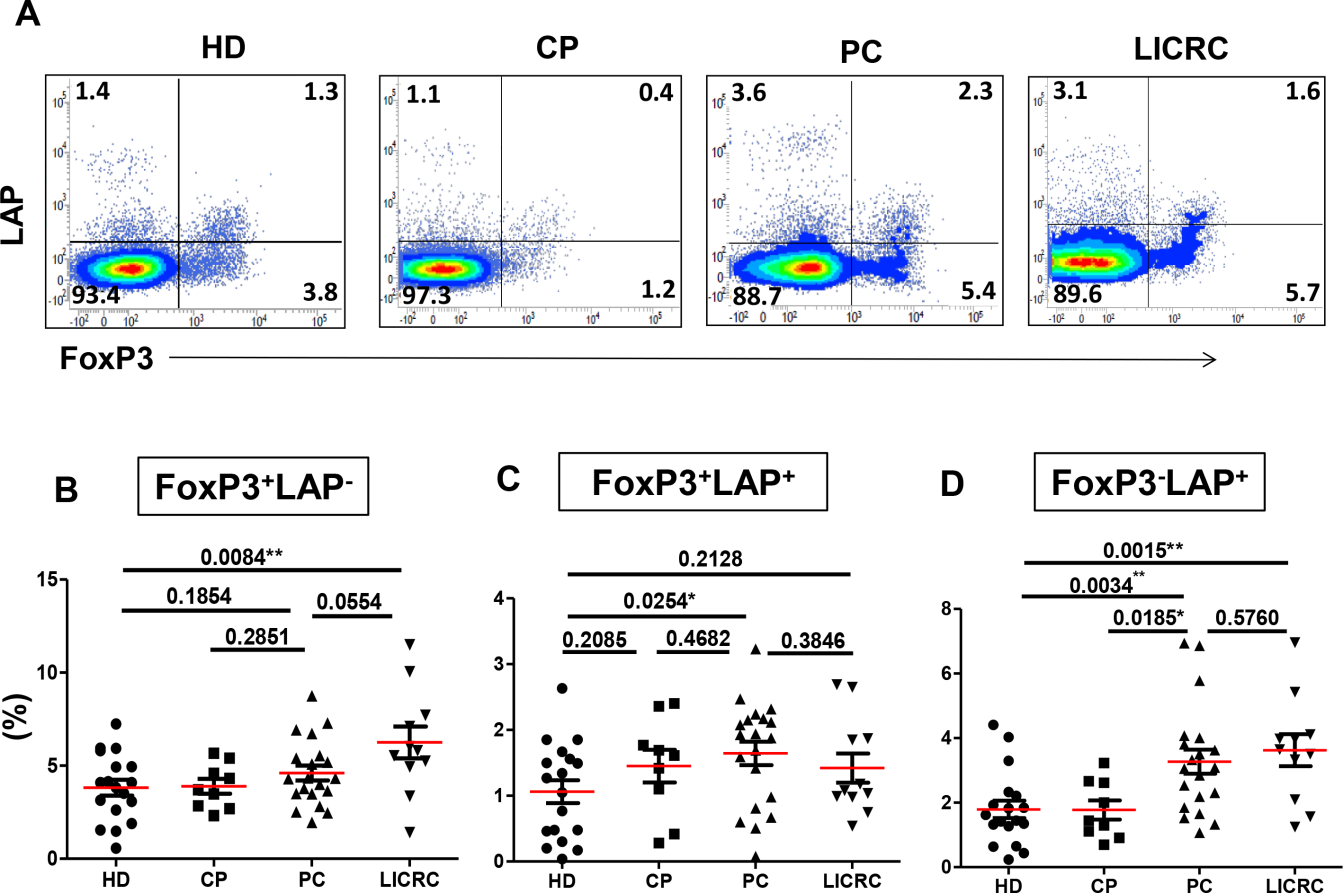
Comparisons between healthy donors and patients for the expression of LAP on activated FoxP3^+/–^ T cell subsets Thawed PBMCs isolated from 18 healthy donors (HD), 9 chronic pancreatitis (CP), 20 pancreatic cancer (PC), and 11 liver metastases from colorectal cancer (LICRC) patients were activated by plate-bound anti-CD3/28 and then stained for surface and intracellular markers. (**A**) Representative flow cytometric plots showing FoxP3 expression against LAP, as gated on CD3^+^CD4^+^T cells from both healthy donors and patients. Scatter plots show the mean percentages ± SEM of FoxP3^+^LAP^−^ (**B**), FoxP3^+^LAP^+^ (**C**) and FoxP3^−^LAP^+^ T cells (**D**).

We further analyzed the levels of LAP^+/–^ and FoxP3^+/–^ CD4^+^ T cell subsets following *in vitro* activation with anti-CD3/28 (Figure 3). We observed similar results to those in the non-activated setting. The FoxP3^+^LAP^−^ Treg subset was significantly expanded only in LICRC (6.25 ± 0.85%) compared with HD (3.82 ± 0.42%) (Figure 3B), and they were higher than PC (4.60 ± 0.40%) although this did not reach significance. The double-positive FoxP3^+^LAP^+^ Treg subset was higher in activated samples (Range in all groups: 1.06–1.64), compared with non-activated samples (Figure 2C and 3C). As expected, the FoxP3^−^LAP^+^ Treg subset was increased in activated cells (Range in all groups: 1.77–3.63), compared with non-activated cells (Range in all groups: 0.6–1.98). Similar to non-activated cells, the FoxP3^−^LAP^+^ subset was expanded in activated cells both in LICRC (3.63 ± 0.50%) and PC (3.27 ± 0.37%), compared with HD (1.79 ± 0.27%) and CP controls (1.77 ± 0.30%) (Figure 3D).

These results emphasize the importance of determining Treg levels by considering different Treg-related markers and not only based on FoxP3 expression as the sole Treg-specific marker.

### FoxP3^+/–^Helios^+^ T cells are expanded in LICRC patients in non-activated and activated settings

**Figure 4:**
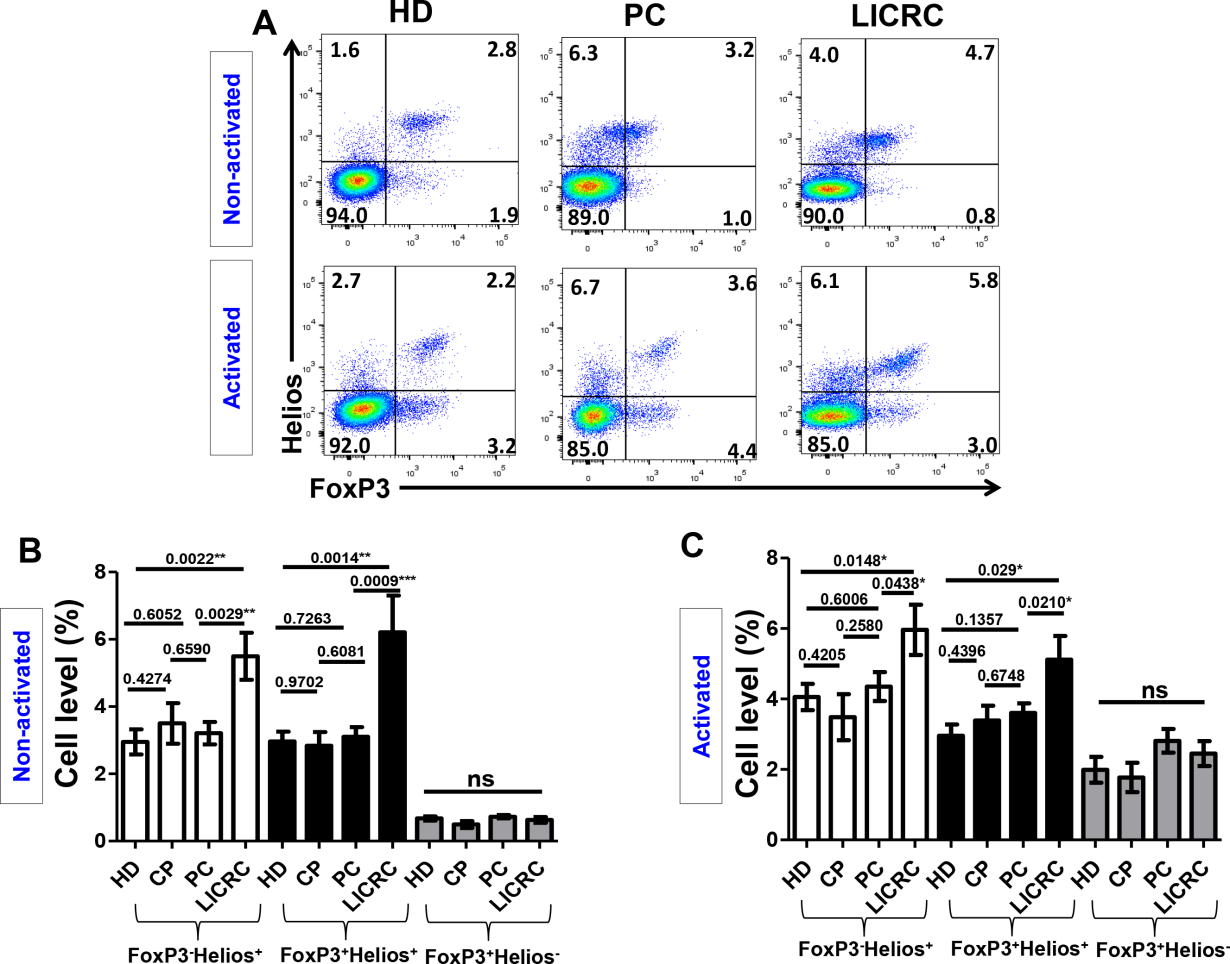
Expression of FoxP3 and Helios on non-activated and activated CD3^+^CD4^+^ T cells (**A**) Representative flow cytometric plots showing the expression of FoxP3 against Helios on healthy donors and cancer patients. (**B**) Bar charts show the mean percentages ± SEM of FoxP3^−^Helios^+^, FoxP3^+^Helios^+^ and FoxP3^+^Helios^−^ T cell subsets in non-activated PBMCs isolated from 14 healthy donors (HD), 7 chronic pancreatitis (CP), 17 pancreatic cancer (PC) and 7 liver metastases from colorectal cancer (LICRC) patients. (**C**) Bar charts show the mean percentages ± SEM of FoxP3^−^Helios^+^, FoxP3^+^Helios^+^ and FoxP3^+^Helios^−^ T cell subsets in activated PBMCs isolated from 18 HD, 9 CP, 20 PC and 11 LICRC patients.

Next, we combined FoxP3 and Helios staining and compared FoxP3^+/–^Helios^+/–^ T cell subpopulations in cells isolated from HD, CP, PC and LICRC in non-activated and activated settings (Figure 4). We previously reported that the expanded FoxP3^+^ Treg subset from peripheral blood of untreated renal cell carcinoma patients and also following IL-2 treatment co-express Helios [27]. In this study, we found that FoxP3^+^Helios^+^ and FoxP3^−^Helios^+^ T cell subsets were significantly higher than FoxP3^+^Helios^−^ Tregs in all subgroups (Figure 4). There was no significant difference in levels of FoxP3^+^Helios^−^ T cells between patients and controls both in activated and non-activated settings (Figure 4B and 4C). However, FoxP3^+^Helios^+^ and FoxP3^−^Helios^+^ T cell subsets were expanded only in peripheral blood of LICRC samples both in non-activated (Figure 4B) and activated cells (Figure 4C), compared with PC and HD (Figure 4B and 4C). There were no significant differences between HD, CP and PC samples in the FoxP3^+/–^Helios^+^ T cell subpopulations. Of interest, the FoxP3^+^Helios^+^ and FoxP3^−^Helios^+^ T cell subsets in LICRC patients were not significantly expanded following activation (Figure 4B and 4C). On the other hand, the FoxP3^+^Helios^−^ subset was expanded following activation (Range in all groups: non-activated: 0.49–0.73% and activated: 1.77–2.45).

### GARP/LAP expression on FoxP3^+/−^Helios^+/−^ T cell subsets in cancer patients, compared with healthy donors

**Figure 5:**
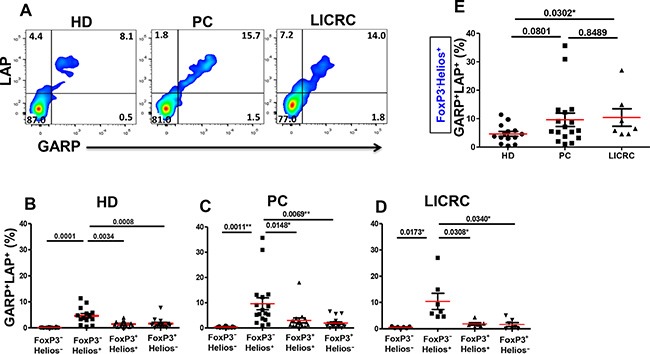
Expression of GARP and LAP on non-activated FoxP3^+/–^Helios^+/–^ T-cell subsets (**A**) Representative flow cytometric plots showing LAP/GARP expression on FoxP3^−^Helios^+^ T-cell subset in non-activated PBMCs from healthy donors (HD) and patients with pancreatic cancer (PC) or liver metastases from colorectal cancer (LICRC). (**B**) Scatter plots showing the mean percentages ± SEM of GARP^+^LAP^+^ cells within FoxP3^+/–^Helios^+/–^ T-cell subsets in non-activated PBMCs isolated from 14 HD (B), 17 PC (**C**) and 7 LICRC patients (**D**). (**E**) Scatter plots comparing the mean percentages ± SEM of GARP^+^LAP^+^ cells within non-activated FoxP3^−^Helios^+^ T-cell subset in HD, PC and LICRC patients.

**Figure 6:**
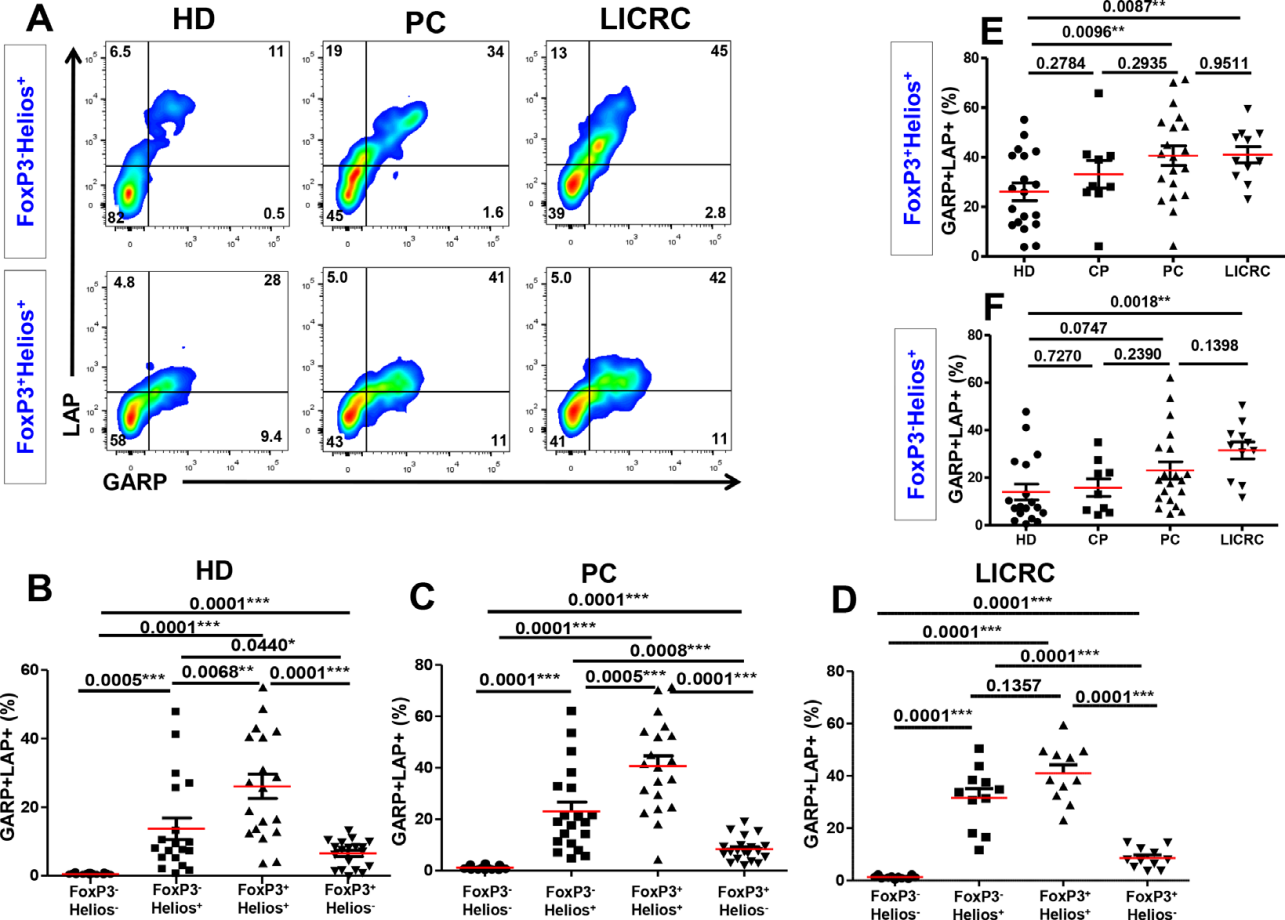
Expression of GARP and LAP on different FoxP3^+/–^Helios^+/–^ T-cell subsets in the activated setting (**A**) Representative flow cytometric plots showing LAP/GARP expression on FoxP3^−^Helios^+^ and FoxP3^+^Helios^+^ T-cell subsets in activated PBMCs from healthy donors (HD), pancreatic cancer (PC) and liver metastases from colorectal cancer (LICRC) patients. (**B**) Scatter plots show the mean percentages ± SEM of GARP^+^LAP^+^ cells within FoxP3^+/–^Helios^+/–^ T-cell subsets in activated PBMCs isolated from 18 HD (B), 20 PC (**C**) and 11 LICRC patients (**D**). Scatter plots comparing the mean percentages ± SEM of GARP^+^LAP^+^ cells within activated FoxP3^+^Helios^+^ (**E**) and FoxP3^−^Helios^+^ (**F**) T-cell subset in HD, CP, PC and LICRC patients.

We further investigated co-expression of LAP and GARP on FoxP3^+/–^Helios^+/–^ T cell subsets in HD, PC and LICRC patients in non-activated (Figure 5) and activated settings (Figure 6). We have recently reported that the only subpopulation that expressed significantly higher levels of GARP/LAP, compared with other subpopulations, was CD4^+^FoxP3^−^Helios^+^ in healthy donors in the non-activated setting (Figure 5B and [26]). Herein, we report similar observations in cells isolated from PC (Figure 5C) and LICRC patients (Figure 5D). Interestingly, levels of CD4^+^FoxP3^−^Helios^+^GARP^+^LAP^+^ Tregs were significantly expanded in LICRC samples (10.41 ± 3.09%, Figure 5E), compared with healthy donors (4.66 ± 0.86%). There was an increase in this Treg subset in PC (9.60 ± 2.36%, Figure 5E) compared with HD, although this did not reach statistical significance (*P =* 0.0801).

Following TCR stimulation, GARP/LAP were up regulated on all T cell subsets (FoxP3^+^Helios^+^, FoxP3^−^Helios^+^ and FoxP3^+^Helios^−^) except the FoxP3^−^Helios^−^ T cell subset (Figure 6B–6D). As we found in HD (Figure 6B and [26]), GARP/LAP were mainly expressed on the FoxP3^+^Helios^+^ T cell subset in activated cells from PC (Figure 6C) and LICRC patients (Figure 6D). Interestingly, the CD4^+^FoxP3^+^Helios^+^GARP^+^LAP^+^ Treg subset was significantly expanded in PC and LICRC samples, compared with healthy donors (Figure 6E). Similar to the non-activated setting, the CD4^+^FoxP3^−^Helios^+^GARP^+^LAP^+^ subset was significantly expanded only in LICRC samples, compared with HD (Figure 6F), and their levels were higher in PC than HD, although this did not reach significance (*P =* 0.0747). GARP/LAP expression on FoxP3^+^Helios^−^ Tregs were significantly lower than their expression on FoxP3^+^Helios^+^ and FoxP3^−^Helios^+^ in healthy donors and cancer patients (Figure 6B–6D). There were no significant differences in GARP/LAP expression on FoxP3^+^Helios^−^ Tregs between HD (6.51 ± 0.92%), PC (8.36 ± 1.04%) and LICRC patients (8.60 ± 1.18%). The potential role of the FoxP3^+^Helios^−^ Treg subset in these cancers could be less significant as they were not expanded in cancer patients, at least in peripheral blood.

### GARP^−/+^LAP^+^ CD4^+^ T cells make IL-10 and their levels are higher in LICRC patients

**Figure 7:**
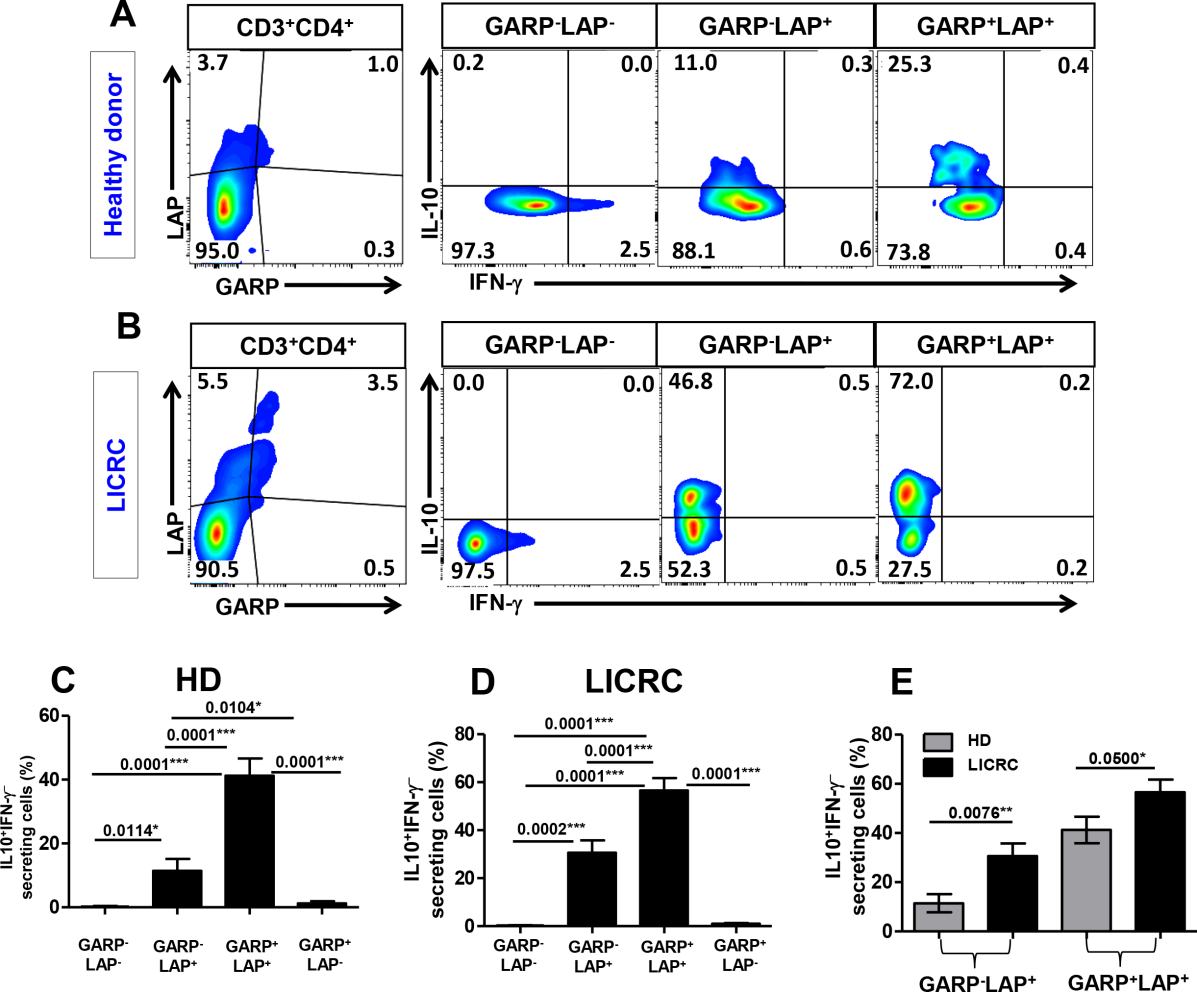
Intracellular cytokine secretion from different GARP^+/–^LAP^+/–^ CD4^+^ T cell subsets Representative flow cytometric plots showing GARP/LAP expression on activated CD3^+^CD4^+^ T cells and levels of IFN-γ and IL-10 secretion from different GARP^+/–^LAP^+/–^ CD4^+^ T cell subsets isolated from peripheral blood of a healthy donor (**A**) and LICRC patient (**B**). Bar charts show the mean percentage ± SEM of IL-10^+^IFN-γ^−^ cells within GARP^+/–^LAP^+/–^ CD4^+^ T cell subsets in PBMCs isolated from 10 healthy donors (**C**) and 10 LICRC patients (**D**). Bar chart comparing the mean percentage ± SEM of IL-10^+^IFN-γ^−^ cells within GARP^−^LAP^+^ and GARP^+^LAP^+^ CD4^+^ T-cell subsets between HD and LICRC patients (**E**).

In order to further define the lineage of LAP and GARP expressing CD4^+^ T cells, we investigated the IL-10 and IFN-γ secretion profile of GARP^+/–^LAP^+/–^ subsets (Figure 7). PBMCs from healthy donors and LICRC patients were activated with anti-CD3/28 in order to induce GARP/LAP expression and stimulate cytokine secretion, followed by addition of Golgi Plug for 4 hours to retain cytokines inside cells. In healthy donors and LICRC patients, the GARP^+^LAP^+^ T cell subset contained the most IL-10 secreting and IFN-γ non-secreting CD4^+^ T cells, defined as IL-10^+^IFN-γ^−^. The GARP^−^LAP^+^ CD4^+^ T cell subset contained a lower level of IL-10^+^IFN-γ^−^ T cells (Figure 7A and 7B). In healthy donors and LICRC samples, the GARP^+^LAP^+^ T cell subsets made significantly higher levels of IL-10 compared to the GARP^−^LAP^+^ T cell subsets. The GARP^+^LAP^−^ and GARP^−^LAP^−^ T cell subsets produced negligible amounts of IL-10 in both HD and LICRC samples.

**Figure 8:**
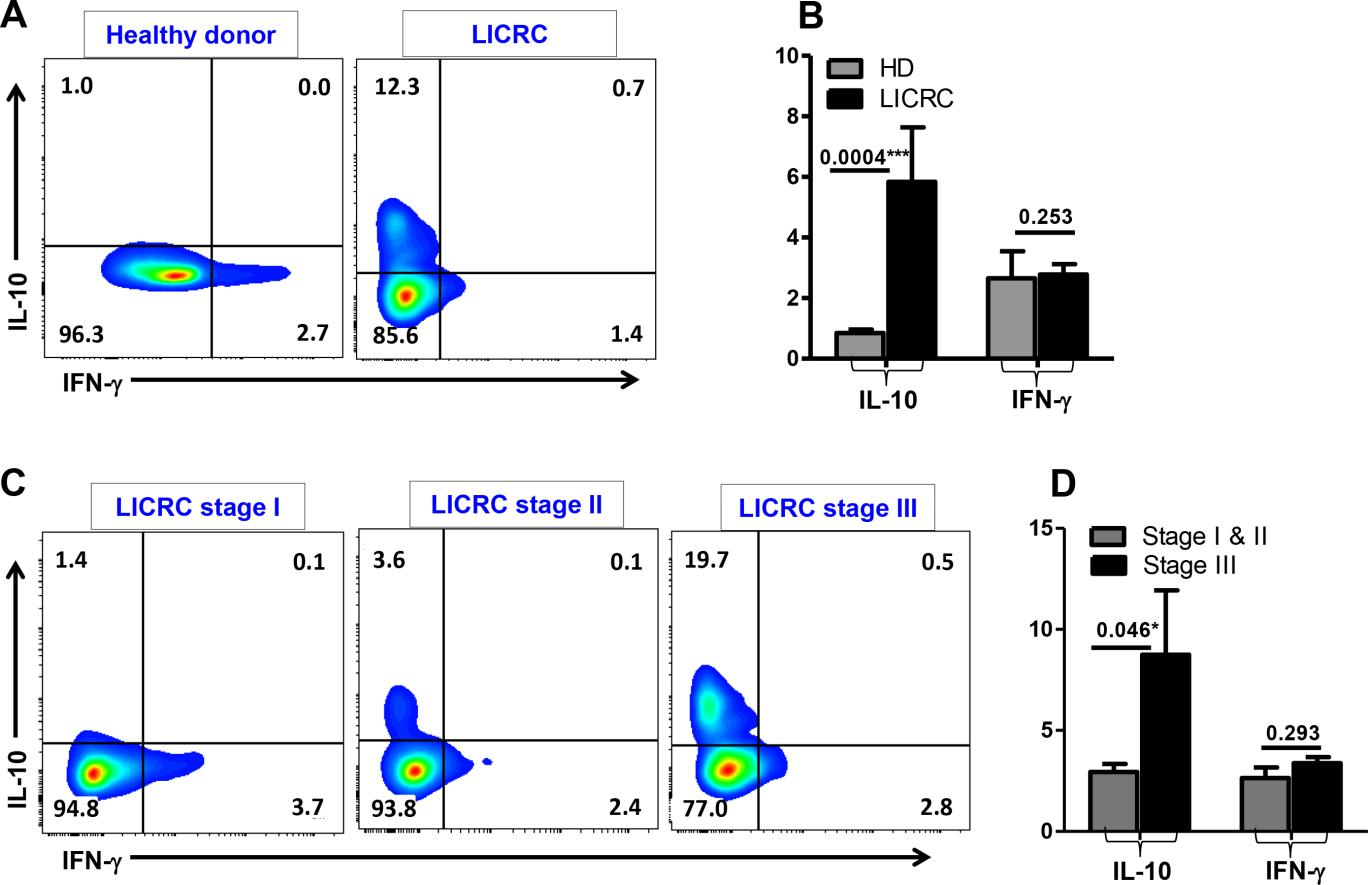
Intracellular cytokine secretion from CD4^+^ T cells Representative flow cytometric plots showing IFN-γ and IL-10 secretion from activated CD3^+^CD4^+^ T cells isolated from peripheral blood of a healthy donor and LICRC patient (**A**), and LICRC patients with TNM staging I, II and III (**C**). Bar chart shows the mean percentage ± SEM of IL-10- and IFN-γ-secreting CD4^+^ T cells in PBMCs isolated from 9 healthy donors and 10 LICRC patients (**B**), and 5 LICRC patients with staging I/II and 5 patients with staging III (**D**).

Interestingly, levels of IL-10-secreting CD4^+^ T cells within GARP^+^LAP^+^ and GARP^−^LAP^+^ subsets were significantly higher in LICRC patients than HD (Figure 7C). The increase in IL-10 secretion in LICRC patients was further confirmed by measuring IL-10 secretion in the whole CD4^+^ T cell population (Figure 8A and 8B), thus confirming the immunosuppressive milieu in cancer patients. When LICRC patients were stratified according to TNM staging, CD4^+^ T cells from LICRC patients with stage III made significantly higher levels of IL-10 than patients with stage I and II (Figure 8C and 8D). Of interest, there was no significant increase in IFN-γ-secreting CD4^+^ T cells between HD and LICRC patients or between LICRC patients with different staging (Figure 8).

## DISCUSSION

In this study, we found that FoxP3^+/–^Helios^+^ Tregs were significantly expanded in the peripheral blood of LICRC patients, compared with healthy donors and PC patients in non-activated and activated settings. Further defining Tregs by expression of GARP and LAP showed that FoxP3^−^LAP^+^ Tregs and activated FoxP3^+/–^Helios^+^GARP^+^LAP^+^Tregs were significantly expanded in cancer patients.

FoxP3^−^LAP^+^ Tregs were identified as a novel suppressive Treg subset in healthy donors where they made up approximately 2% of the circulating CD4^+^ T cell compartment [28]. Several groups have since characterized highly suppressive FoxP3^+/–^LAP^+^ Tregs in healthy donors and cancer patients [24, 29–31]. The nature of GARP and LAP expression on T cells in cancer remains uncertain. In patients with head and neck squamous cell carcinoma, highly suppressive CD4^+^CD39^+^GARP^+^LAP^+^ Tregs were expanded following chemoradiation therapy [32]. In hepatocellular carcinoma and ovarian cancer, GARP-expressing FoxP3^+^ Tregs were expanded in the peripheral blood and ascites, respectively [6, 33]. In colorectal cancer patients, FoxP3^−^LAP^+^ Tregs have been correlated with cancer progression and were reported to be expanded in the peripheral blood of patients with tumor metastases, compared with healthy donors and non-metastatic patients [29–31]. Another study reported that FoxP3^−^LAP^+^ TI Tregs isolated from tumour tissue exhibited potent *in vitro* suppressive activity mediated by TGF-β and IL-10, and were up to 50-fold more suppressive than ‘conventional' FoxP3^+^ Tregs [31]. In this study, we confirmed the presence of a significant peripheral blood FoxP3^−^LAP^+^ Treg subset, and the majority of these cells co-expressed GARP and Helios.

We found that FoxP3^−^Helios^+^GARP^+^LAP^+^ Tregs were significantly expanded in LICRC patients, while FoxP3^+^Helios^+^GARP^+^LAP^+^ Tregs were significantly increased in PC and LICRC patients. The selective co-expression of GARP/LAP with Helios is intriguing. Helios^+^ Tregs have been shown to overexpress TGF-β and to exhibit potent TGF-β mediated suppressive activity [21]. While we did not test the suppressive function of Helios^+/–^ T cell subsets, GARP/LAP co-expression could indicate a robust TGF-β mediated suppressive mechanism. There have been limited investigations into GARP/LAP expression with FoxP3 and Helios. GARP and FoxP3 have been proposed to form a positive feedback loop, although more recent work showed that regulation of GARP is independent of FoxP3 and GARP was also shown not to correlate with Helios expression in FoxP3^+^ T cells [34, 35]. It remains to be confirmed if there is any mechanistic link between GARP/LAP expression and Helios expression.

The increased levels of FoxP3^+/−^Helios^+^GARP^+^LAP^+^ Tregs in LICRC patients could be attributed to the advanced metastatic stage of these patients, and the associated tumour-mediated immunosuppression that could be expected. In the activated setting, FoxP3^+^Helios^+^GARP^+^LAP^+^ Tregs outnumbered FoxP3^−^Helios^+^GARP^+^LAP^+^ Tregs in all samples. The underlying reasons for this are not immediately clear. However, FoxP3^+^Helios^+^ Tregs have been shown to preferentially expand *in vivo* compared to FoxP3^+^Helios^−^ [27, 36].

Interestingly, GARP and LAP were expressed at relatively high levels on the FoxP3^−^Helios^+^ Treg subset in the non-activated setting, and they were expanded in LICRC patients (HD: ∼5%, LICRC: ∼10%). This FoxP3^−^Helios^+^GARP^+^LAP^+^ Treg subset has not been previously described and could represent the novel FoxP3^−^LAP^+^ Treg subset described in previous studies [28, 31], emphasizing the importance of Helios as a Treg marker. However, the suppressive ability of this Treg subset will need to be confirmed in functional studies.

We also found that cells from LICRC patients secrete more IL-10, which is confined to the GARP^+/–^LAP^+^ T-cell compartments. Interestingly, LICRC patients with higher TNM staging had higher levels of IL-10-secreting CD4^+^ T cells. This increase in IL-10 might be indicative of increased Treg activity, especially given the advanced metastatic stage of LICRC patients. Our findings support the role of GARP/LAP as markers of IL-10-secreting Tregs, while co-expression of GARP/LAP also infer the potential of a TGF-β mediated suppressive mechanism of these cells.

Our data support the role of GARP and LAP as markers of Tregs, and potentially novel immunotherapy targets. GARP-blocking antibodies have already been developed and shown to inhibit Treg activity in a xenogeneic model of graft-versus-host-disease [37]. In the absence of further clinical and functional data, we cannot comment on the exact nature and origin of FoxP3^+/–^Helios^+^GARP^+^LAP^+^ Tregs whether thymic or peripheral or even induced in the tumour microenvironment. Investigating the correlation between the levels of these Treg subsets and disease prognosis was not possible due to the relatively small number of samples, and it is imperative to investigate this correlation. Further studies are required to confirm the nature, origin and clinical impact of the FoxP3^+/–^Helios^+^GARP^+^LAP^+^ T cell subsets identified in this study. It will also be important to elucidate the role of Helios expression in Tregs and T cells, whether as an activation marker or as part of a suppressive mechanism.

Taken together, our results indicate that studies investigating Tregs in different pathological settings should consider different Treg-related markers such as GARP, LAP, Helios, and not only FoxP3 as a sole Treg-specific marker. Understanding the role and contribution of specific Treg subsets in various pathological settings will enable the development of effective immunotherapies, targeting only the most ‘pathological' or suppressive Treg subsets as opposed to systemic therapies.

## MATERIALS AND METHODS

### Collection of blood samples

The research protocol was approved by the UK National Research Ethical Committee, Salford Research Ethics Committee and the Local Research and Development Departments. Written consent was obtained from all patients and healthy donors before blood collection. Samples were collected from patients with chronic pancreatitis (CP, *n =* 9), malignant pancreatic cancer (PC, *n =* 20) or liver metastases from colorectal cancer (LICRC, *n =* 11) at the North Manchester General Hospital, UK. Table 1 shows the characteristic features of all patients in this study. In addition, blood samples were collected from healthy donors (HD) as controls. Blood samples were collected in a 50 ml Falcon tube containing 200 μl (1000 IU/ml) heparin.

### Cell isolation and preparation

Peripheral blood mononuclear cells (PBMCs) were isolated from whole blood using Ficoll-Hypaque (Sigma-Aldrich, UK) density gradient centrifugation. PBMCs were then frozen at 5–10 × 10^6^ cells/ml in cryovials in 1 ml of freezing media (50% FCS, 40% RPMI-1640 and 10% DMSO) and stored in liquid nitrogen (LN) for later use. Trypan blue was used for PBMC viability testing and counting.

### *In vitro* T cell culture

PBMCs were thawed and suspended at 2 × 10^6^ cells/well in 2 ml complete medium [RPMI-1640 supplemented with L-glutamine 2 mM, 10% FCS, Streptomycin 100 μg/ml and Penicillin 100 Units/ml]. 24-well non-treated culture plates were pre-coated with plate-bound 2 μg/ml anti-CD3 antibody (OKT3 clone, eBioscience, Hatfield, UK) and 2 μg/ml anti-CD28 antibody (CD28.2 clone, eBioscience) for 2.5 hours at 37°C. PBMCs were either plated as ‘non-activated' in non-coated wells or ‘activated' in pre-coated wells. Plated cells were incubated for 18–20 hours in a humidified incubator at 37°C and 5% CO_2_. Cells were collected and blocked for FcR with IgG from human serum (Sigma-Aldrich), ready for staining and flow cytometric analysis.

### Cell staining and flow cytometric analysis

*Surface staining:* Cells were then washed and labeled for surface markers: mouse anti-human CD4-PerCP-Cy5.5 (RPA-T4 clone, eBioscience), mouse anti-human CD3-APC-H7 (SK7 clone, BD Biosciences, Oxford, UK), mouse anti-human GARP-APC (7B11 clone, BD Biosciences), and mouse anti-human LAP-PE (TW4–2F8 clone, BD Biosciences). *Intracellular staining:* Fixation, permeabilization and flow cytometry buffers were from eBioscience or BD Biosciences and prepared as per the manufacturer's instructions. Following staining for surface markers, cells were fixed and permeabilized at 4°C for 45 minutes using fixation/permeabilization buffer. Cells were then blocked for 15 minutes using rat serum (eBioscience) and mouse serum (Sigma-Aldrich) before staining with rat anti-human FoxP3-PE-Cy7 (PCH101 clone, eBioscience) and Armenian hamster anti-mouse/human Helios-FITC (22F6 clone, Biolegend, Cambridge, UK) for 30 minutes at 4°C. Following two further permeabilization washes using permeablization buffer, cells were resuspended in flow cytometry buffer. *Cytokine detection:* Thawed PBMCs were plated in complete medium in a 24-well non-treated culture plate pre-coated with 2 μg/ml anti-CD3 and 2 μg/ml anti-CD28. To investigate IFN-γ and IL-10 release from GARP^+/–^LAP^+/–^ Treg subpopulations, cells were incubated for 24 hours at 37°C and 5% CO_2_. 1 μg/ml Golgi Plug (BD Biosciences) was added for the last 4 hours of activation. Cells were first stained for surface markers using mouse anti-human CD4-PerCP-Cy5.5, mouse anti-human CD3-APC-H7, mouse anti-human GARP-APC, and mouse anti-human LAP-PE. For intracellular cytokines, cells were subsequently fixed, permeabilized and blocked using mouse serum before staining with mouse anti-human IL-10-FITC (BT-10 clone, eBioscience) and mouse anti-human IFN-γ-PE-Cy7 (4S.B3 clone, BD Pharmingen, BD Biosciences, UK).

Flow cytometric data was acquired on FACSVerse or FACSCanto II flow cytometers (BD Biosciences, USA). Data analysis was performed using BD FACSuite or FlowJo 10.0.8r1 software.

### Statistical analysis

Statistical analysis was performed using GraphPad Prism 5.0 software (GraphPad Software, USA). Paired *T* test or unpaired/Mann-Whitney tests were used to examine for differences within groups or between groups, respectively. *P* value ≤ 0.05 was considered statistically significant. The data are presented as means ± SEM.
